# Maternal smoking cessation in the first trimester still poses an increased risk of attention-deficit/hyperactivity disorder and learning disability in offspring

**DOI:** 10.3389/fpubh.2024.1386137

**Published:** 2024-07-16

**Authors:** Qiu Li, Xiaotang Cai, Hui Zhou, Dan Ma, Na Li

**Affiliations:** ^1^Department of Rehabilitation Medicine, West China Second University Hospital, Sichuan University, Chengdu, Sichuan, China; ^2^Key Laboratory of Birth Defects and Related Diseases of Women and Children (Sichuan University), Ministry of Education, Chengdu, Sichuan, China

**Keywords:** maternal smoking cessation, attention-deficit/hyperactivity disorder, learning disability, offspring, pregnancy

## Abstract

**Background:**

Studies have found maternal smoking during pregnancy was linked to attention-deficit/hyperactivity disorder (ADHD) risk. It is unclear if maternal smoking cessation during pregnancy lowers ADHD and learning disability (LD) risk in offspring. This study aimed to explore the associations between maternal smoking cessation during pregnancy and ADHD and LD risk in offspring.

**Methods:**

Data from the National Health and Nutrition Examination Survey 1999–2004 (8,068 participants) were used. Logistic regression was used to analyze the associations between maternal smoking and smoking cessation during pregnancy and ADHD and LD risk in offspring.

**Results:**

Compared to non-smokers’ offspring, maternal smoking during pregnancy increased the risk of ADHD (odds ratios [OR] = 2.07, 95% confidence interval [CI]: 1.67–2.56) and LD (OR = 1.93, 95% CI: 1.61–2.31) in offspring, even if mothers quit smoking later (OR_ADHD_ = 1.91, 95%CI_ADHD_: 1.38–2.65, OR_LD_ = 1.65, 95%CI_LD_: 1.24–2.19). Further analysis of the timing of initiation of smoking cessation during pregnancy revealed that, compared to non-smokers’ offspring, maternal quitting smoking in the first trimester still posed an increased risk of ADHD (OR = 1.72, 95% CI: 1.41–2.61) and LD (OR = 1.52, 95% CI: 1.06–2.17) in offspring. Maternal quitting smoking in the second or third trimester also had a significantly increased risk of ADHD (OR = 2.13, 95% CI: 1.26–3.61) and LD (OR = 1.82, 95% CI: 1.16–2.87) in offspring. Furthermore, maternal smoking but never quitting during pregnancy had the highest risk of ADHD (OR = 2.17, 95% CI: 1.69–2.79) and LD (OR = 2.10, 95% CI: 1.70–2.58) in offspring. Interestingly, a trend toward a gradual increase in the risk-adjusted OR for ADHD and LD risk was observed among the three groups: maternal quitting smoking in the first trimester, maternal quitting smoking in the second or third trimester, and maternal smoking but never quitting.

**Conclusion:**

Maternal smoking cessation in the first trimester still poses an increased risk of ADHD and LD in offspring. Furthermore, it seems that the later the mothers quit smoking during pregnancy, the higher the risk of ADHD and LD in their offspring. Therefore, early intervention of maternal smoking in preconception and prenatal care is vital for offspring neurodevelopment.

## Introduction

Neurodevelopmental disorders are a group of conditions with onset in the developmental period characterized by developmental deficits that produce impairments in personal, social, academic, or occupational functioning (1). Among neurodevelopmental disorders, attention-deficit/hyperactivity disorder (ADHD) and learning disability (LD) are common in children. ADHD is developmentally inappropriate and impairing inattention, motor hyperactivity, and impulsivity, and these difficulties usually persist into adulthood (2). LD is a state of learning difficulty that is exhibited specifically in reading, writing, spelling, expression, and computation (1). The global community prevalence of ADHD ranges from 2 to 7%, with an average of approximately 5% (3). The reported prevalence is 9.4% in U.S. children and adolescents (4), 18.1% in Tunisian adolescents (5), 8.8% in Nigerian children (6) and 6.26% in Chinese children and teenagers (7). As for LD, the prevalence of specific learning disorder in academic areas like reading, writing, and math ranges from 5 to 15% among school-aged children across languages and cultures (1). It is widely accepted that genetic and environmental factors or a complex interplay between them contribute to developing ADHD and LD (1, 2, 8). However, the specific factors and their respective roles remain unidentified. In addition to the critical role of genetic factors, several environmental factors may also contribute to ADHD and LD, such as environmental toxins, psychosocial adversity, and prenatal and perinatal factors (9, 10). The life-course approach to disease emphasizes the importance of the intrauterine environment in preventing future disease, and the preconception period is seen as a critical period where interventions can provide long-term benefits (11). The Developmental Origins of Health and Disease (DOHaD) hypothesis highlights the potential impact of adverse environmental factors experienced by mothers on the health of their offspring, which may not be limited to childhood and adolescence but may persist into adulthood (12). Several studies have investigated prenatal and perinatal factors such as low birth weight (13), preterm birth (14), exposure to environmental toxins (15) and maternal smoking (15). Studies have revealed that smoking through harmful substances in tobacco, such as nicotine, may negatively affect neurological development (16, 17).

Maternal smoking during pregnancy is a detrimental and hazardous habit that is thought to negatively affect the health of both the mother and the developing fetus. The global prevalence of smoking during pregnancy was estimated to be 1.7%, with the highest prevalence in Ireland (38.4%), Uruguay (29.7%), and Bulgaria (29.4%) (18). Notably, over half (52.9%) of the women continued to smoke daily during pregnancy, varying from 30.6% in the European region to 79.6% in the Western Pacific region (18). This means that a certain percentage of babies were exposed to smoking prenatally. Maternal smoking during pregnancy is associated with many detrimental infant health outcomes, such as preterm birth (19), low birth weight (20), and congenital developmental malformations (21). Additionally, maternal smoking during pregnancy is associated with neurodevelopmental and behavioral consequences in the offspring. Epidemiological studies indicate that maternal smoking during pregnancy increases the risk of ADHD (22–28) and LD (29, 30) in offspring. Biederman et al. (31) showed that maternal smoking during pregnancy was associated with a more severe form of ADHD in children, characterized by more severe clinical and neuropsychological manifestations. Similarly, a birth cohort study revealed that maternal smoking during pregnancy was associated with a slight decline in offspring academic performance, with the potential effects persisting into adolescence (32). Animal studies have also found that prolonged nicotine exposure during pregnancy was associated with reduced attention, increased hyperactivity, and learning and memory deficits in offspring (33–35). Alkam et al. (36) revealed that nicotine at any point of the gestational and perinatal period impaired emotional behaviors in offspring. Even though some studies suggest that this association is explained by unmeasured genetic or environmental confounding (37, 38), both experimental animal and human studies have shown that tobacco smoke contains numerous toxic components, including nicotine and carbon monoxide, which can lead to changes in enzyme and hormone levels, as well as in the expression of genes, microRNAs, and proteins early in a child’s life (39). These changes may be associated with neurobehavioral and cognitive deficits in the offspring (40–42).

Given the hazards of smoking, some studies have focused on the relationship between smoking cessation and perinatal outcomes. Population-based studies have shown that quitting smoking during the first trimester of pregnancy decreases the risk of small for gestational age (SGA) (43, 44), stillbirth (45), and preterm (46). Besides, other studies have also focused on the relationship between maternal smoking cessation during pregnancy and the risk of health problems in offspring. A meta-analysis suggested that maternal smoking cessation could potentially reduce childhood overweight and obesity risks (47). Piper et al. (48) showed that children whose mothers quit smoking during pregnancy had significantly fewer attention and externalizing problems than those whose mothers continued smoking. Heinonen et al. (49) found that heavy smoking before pregnancy was associated with lower cognitive abilities in offspring, even if the mother had quit smoking before pregnancy. However, specific evidence on whether maternal smoking cessation during pregnancy can mitigate ADHD and LD risks in offspring is currently insufficient; furthermore, studies on the effects of smoking cessation timing are lacking. Further studies are needed to clarify the relationship between maternal smoking cessation during pregnancy and these particular developmental issues.

Therefore, this study, based on the National Health and Nutrition Examination Survey (NHANES), aimed to evaluate the relationship between maternal smoking habits during pregnancy and the risk of ADHD and LD in offspring. Within this scope, we pursued two objectives. First, we assessed the smoking and quitting status of mothers during pregnancy in children with ADHD and LD. Second, we attempted to understand the relationship between maternal smoking and quitting during pregnancy and the risk of these two diseases.

## Materials and methods

### Study population

**Figure 1:**
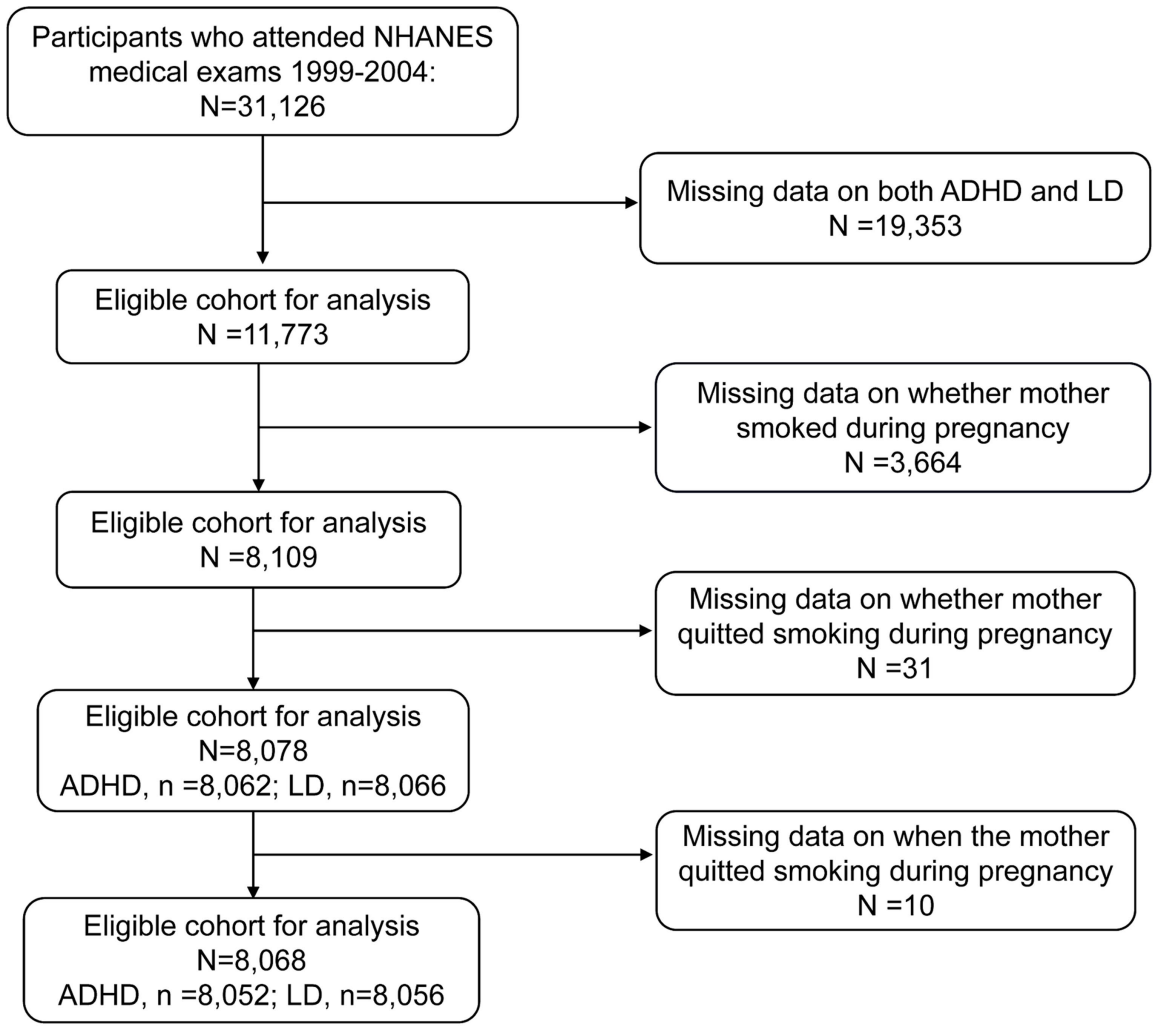
Eligible participants and those included in the analysis of the associations between maternal smoking history and the risk of ADHD and LD in offspring in the U.S. population. ADHD, attention-deficit/hyperactivity disorder; LD, learning disability; NHANES, National Health and Nutrition Examination Survey.

The data used in this study were derived from NHANES, a comprehensive survey conducted by the National Center for Health Statistics (NCHS). The NHANES gathers information on individuals’ health and nutritional status in the U.S. This survey employs a complex, multistage, stratified probability cluster design and represents the non-institutionalized civilian population. The NHANES data are released to the public in 2-year cycles. Detailed survey procedures and consent documents of the NHANES are available on the NCHS website^1^. The NCHS Research Ethics Review Board approved these NHANES cycles. All participants provided written informed consent. Data on ADHD and LD outcomes were only available for the 1999–2004 cycle; therefore, we obtained publicly available NHANES data generated through surveys conducted in 1999–2004, including data from 31,126 participants. Using unique survey participant identifiers, we combined information on participants’ characteristics with details from their questionnaires. For maternal smoking and smoking cessation, data were only available for offspring aged 0–15 years, and the age ranges assessed for ADHD and LD were 4–19 and 4–15 years, respectively. Of these participants, 19,353 had neither ADHD nor LD data. Furthermore, 3,664 children did not have information on whether their mothers smoked during pregnancy and were therefore excluded from the analysis. Meanwhile, those without information on whether smoking cessation during pregnancy or the starting time of smoking cessation were also excluded. Ultimately, the final sample size included in the analysis was 8,068 children. Among them, 8,052 participants had available data on ADHD, and 8,056 participants had available data on LD. Figure 1 shows the participant inclusion and exclusion process in a flowchart.

### Assessment of maternal smoking habits during pregnancy and neurodevelopmental outcomes in offspring

This information was based on parental or guardian responses to the NHANES interview question. To determine whether the mother smoked during pregnancy, the following question was asked: “Did survey participant (SP) NAME’s biological mother smoke at any time while she was pregnant with him/her?” We defined a yes answer to this question as the mother smoking during pregnancy (Smoker) and a no answer as the mother not smoking during pregnancy (Non-smoker). Similarly, to determine whether the mother quit smoking during pregnancy, the question asked was: “At any time during the pregnancy, did SP NAME’s biological mother quit or refrain from smoking for the rest of the pregnancy?” We defined a yes answer to this question as if the mother smoked during pregnancy but quit later (Smoke but quit) and a no answer as if the mother smoked during pregnancy but never quit (Smoke but never quit). To gather more specific information about when the mother quit smoking during pregnancy, the question asked was: “About what month of the pregnancy did SP NAME’s biological mother stop smoking?” This response was a specific month, with nine options depending on the number of months of pregnancy, corresponding to the months from January to September. We defined quitting smoking at ≤3 months of pregnancy as quitting smoking in the first trimester (Quit in the first trimester), 4–6 months of pregnancy as quitting smoking in the second trimester (Quit in the second trimester), and quitting smoking after ≥7 months of pregnancy as quitting smoking in the third trimester (Quit in the third trimester). To assess the neurodevelopmental outcomes, including ADHD and LD, the parents/guardian were asked, “Has a doctor or health professional ever told you/SP that you/s/he/SP had attention deficit disorder?” and “Has a representative from a school or a health professional ever told you/SP that s/he/SP had a learning disability?” The answers to these questions were “yes” or “no.” Based on the responses obtained, the children were categorized into the no-ADHD (individuals without parental or guardian reports of ADHD), yes-ADHD (individuals with parental or guardian reports of ADHD), no-LD (individuals without parental or guardian reports of LD), yes-LD (individuals with parental or guardian reports of LD) groups.

### Other covariables

Some covariables were assessed as potential confounders, including age, gender, race, poverty income ratio (PIR), birth weight, and NHANES cycle. The information regarding these covariables was gathered through face-to-face interviews. Racial categories were “Mexican American,” “Other Hispanic,” “Non-Hispanic white,” “Non-Hispanic Black,” and “Other race-Including-Multi-Racial.” Birth weight data, initially recorded in pounds and ounces, were converted into grams to ensure consistency in the analysis. The NHANES also collected data on whether a child’s birth weight was less than 2,500 g and whether it was more than 4,100 g. Birth weight data were based on parents/guardians’ answers to the following questions: “How much did SP NAME weigh at birth?”, “Did SP NAME weigh more than 5–1/2 lbs. (2,500 g), or less than 5–1/2 lbs. (2,500 g)?,” and “Did (SP NAME) weigh more than 9 lbs. (4,100 g), or less than 9 lbs. (4,100 g)?”

### Statistical analysis

Continuous variables are expressed as medians (interquartile ranges [IQR]), while categorical variables are described as frequencies and percentages. Based on the clinical significance, age and birth weight were transformed into categorical variables for subsequent regression analysis. Specifically, the age variable was categorized as 4–7, 8–11, and 12–15 years. The birth weight variable was divided into <2,500, 2,500–4,100, and ≥4,100 g. Due to missing values in the PIR variable for 682 participants, the median of nearby points was used to replace the missing values to ensure that the sample size was not reduced. For the birth weight variable, 64 participants had missing data and were treated as a separate group in the analysis. The Chi-Square and Mann–Whitney tests were used for comparisons between groups. To analyze the associations between maternal smoking habits during pregnancy and the risk of ADHD and LD in offspring, we used the following three models: Crude Model: No adjustment for variables; Model I: adjusted for gender, age, race, and PIR; and Model II: adjusted for all underlying covariates including age, gender, race, PIR, birthweight, and NHANES cycle. For the sensitivity analysis, we excluded participants without PIR data in regression analysis. Statistical significance was set at *p* < 0.05. SPSS (SPSS 26.0, Inc., United States) and R software (version 4.3.1) were used for statistical analysis. GraphPad Prism software (version 8.0) was used to plot graphs.

## Results

### Study population characteristics

We categorized the children into different groups: no-ADHD: individuals without ADHD; yes-ADHD: individuals with ADHD; no-LD: individuals without LD; yes-LD: individuals with LD. The demographic characteristics between no-ADHD and yes-ADHD groups and between no-LD and yes-LD groups are shown in Table 1. Significant statistical differences were observed in gender, age, and race in no-ADHD and yes-ADHD groups (*p* < 0.001). A similar trend was observed in no-LD and yes-LD groups. In terms of gender composition, the proportion of males in the yes-ADHD group was higher than that in the no-ADHD group (73.2% vs. 47.5%), and the ratio of males in the yes-LD group was higher than that in the no-LD group (61.8% vs. 47.7%). Regarding age, the median ages for the no-ADHD and yes-ADHD groups were 11 (7–13) years and 12 (9–13) years, respectively, and the median ages for the no-LD and LD groups were 10 (7–13) and 12 (9–13) respectively. The no-LD and yes-LD groups had statistically significant differences in PIR and birth weight (*p* < 0.05).

**Table 1:** Characteristics of children by developmental outcomes for study participants.

Characteristic	ADHD				LD			
	No (*N* = 7,540)	Yes (*N* = 522)	Test	*P*	No (*N* = 7,223)	Yes (*N* = 843)	Test	*P*
Age (years) Median (IQR)	11 (7–13)	12 (9–13)	*Z* = −7.01	<0.001	10 (7–13)	12 (9–13)	*Z* = −7.83	<0.001
Age (years), *n* (%)								
4–7	2,231 (29.6)	58 (11.1)	*Z* = −7.74	<0.001	2,167 (30)	124 (14.7)	*Z* = −8.43	<0.001
8–11	2,036 (27)	175 (33.5)			1,947 (27)	262 (31.1)		
12–15	3,273 (43.4)	289 (55.4)			3,109 (43)	457 (54.2)		
Gender								
Female	3,959 (52.5)	140 (26.8)	*χ*^2^ = 128.88	<0.001	3,780 (52.3)	322 (38.2)	*χ*^2^ = 60.36	<0.001
Male	3,581 (47.5)	382 (73.2)			3,443 (47.7)	521 (61.8)		
Race								
Mexican American	2,600 (34.5)	89 (17)	*χ*^2^ = 75.92	<0.001	2,461 (34.1)	227 (26.9)	*χ*^2^ = 24.36	<0.001
Other Hispanic	315 (4.2)	25 (4.8)			298 (4.1)	44 (5.2)		
Non-Hispanic Black	2,359 (31.3)	187 (35.8)			2,242 (31)	304 (36.1)		
Non-Hispanic White	1,946 (25.8)	199 (38.1)			1,904 (26.4)	242 (28.7)		
Other race-Including-Multi-Racial	320 (4.2)	22 (4.2)			318 (4.4)	26 (3.1)		
PIR, Median (IQR)	1.5 (0.8–2.8)	1.7 (0.9–3)	*Z* = −0.92	0.356	1.5 (0.8–2.9)	1.2 (0.7–2.3)	*Z* = −6.97	<0.001
Birth weight (g), *n* (%)								
<2,500	707 (9.4)	61 (11.7)	*χ*^2^ = 6.17	0.104	646 (8.9)	122 (14.5)	*χ*^2^ = 28.12	<0.001
2,500–4,100	6,230 (82.6)	411 (78.7)			5,981 (82.8)	665 (78.9)		
> = 4,100	542 (7.2)	47 (9)			539 (7.5)	50 (5.9)		
Miss	61 (0.8)	3 (0.6)			57 (0.8)	6 (0.7)		
NHANES cycle, *n* (%)								
1999–2000	2,509 (33.3)	167 (32)	*χ*^2^ = 0.48	0.788	2,406 (33.3)	273 (32.4)	*χ*^2^ = 0.41	0.814
2001–2002	2,668 (35.4)	185 (35.4)			2,555 (35.4)	298 (35.3)		
2003–2004	2,363 (31.3)	170 (32.6)			2,262 (31.3)	272 (32.3)		

Data was shown as number (percentage) or Median (IQR). The Chi-Square test and Mann–Whitney test were used in the analysis. ADHD, attention-deficit/hyperactivity disorder; LD, learning disability; PIR, poverty income ratio; NHANES, National Health and Nutrition Examination Survey; IQR, interquartile range.

### Comparison of maternal smoking status during pregnancy between individuals without ADHD and those with ADHD and between those without LD and those with LD

As shown in Table 2, compared to the no-ADHD group, the yes-ADHD group had a higher ratio of mothers who smoked during pregnancy (28.2% vs. 13.4%). Similarly, compared to the no-LD group, the yes-LD group had a higher ratio of mothers who smoked during pregnancy (25.5% vs. 13.1%).

**Table 2:** Comparison of the maternal smoking status during pregnancy at different developmental outcomes of offspring.

Mother’s smoking history	ADHD				LD			
	No (*N* = 7,540)	Yes (*N* = 522)	Test	*P*	No (*N* = 7,223)	Yes (*N* = 843)	Test	*P*
Non-smoker	6,527 (86.6)	375 (71.8)	*χ*^2^ = 85.94	<0.001	6,276 (86.9)	628 (74.5)	*χ*^2^ = 94.03	<0.001
Smoker	1,013 (13.4)	147 (28.2)			947 (13.1)	215 (25.5)		

Data was shown as number (percentage). A Chi-Square test was used in the analysis. ADHD, attention-deficit/hyperactivity disorder; LD, learning disability.

### Associations between maternal smoking habits during pregnancy and the risk of ADHD and LD in offspring

To assess associations between maternal smoking habits during pregnancy and the risk of ADHD and LD in offspring, we first examined the overall risk of smoking for the two diseases. As shown in Supplementary Table S1, in Model II (adjusted for age, gender, race, PIR, birth weight, and NHANES cycle), maternal smoking during pregnancy increased the risk of ADHD (OR = 2.07, 95% CI: 1.67–2.56) and LD (OR = 1.93, 95% CI: 1.61–2.31) in offspring compared to non-smokers’ offspring. Second, we analyzed the risk of quitting smoking for these two diseases. As shown in Table 3, in Model II (adjusted for age, gender, race, PIR, birth weight, and NHANES cycle), maternal smoking during pregnancy but quitting later still increased the risk of ADHD (OR = 1.91, 95% CI: 1.38–2.65) and LD (OR = 1.65, 95% CI: 1.24–2.19) in offspring compared to non-smokers’ offspring.

**Table 3:** Association between maternal smoking habits during pregnancy and neurodevelopmental outcomes of offspring.

	Crude Model		Model I		Model II	
		OR (95% CI)	*P*	OR (95% CI)	*P*	OR (95% CI)	*P*
ADHD							
Mother’s smoking habits during pregnancy							
Non-smoker		Reference		Reference		Reference	
Smoke but quit		2.25(1.64,3.08)	<0.001	1.94(1.40,2.69)	<0.001	1.91(1.38,2.65)	<0.001
Smoke but never quit		2.69(2.12,3.41)	<0.001	2.17(1.69,2.79)	<0.001	2.16(1.68,2.78)	<0.001
LD							
Mother’s smoking habits during pregnancy							
Non-smoker		Reference		Reference		Reference	
Smoke but quit		1.82(1.38,2.39)	<0.001	1.69(1.28,2.25)	<0.001	1.65(1.24,2.19)	0.001
Smoke but never quit		2.55(2.09,3.11)	<0.001	2.18(1.77,2.68)	<0.001	2.10(1.70,2.58)	<0.001

Crude model adjusts for none; Model I adjusts for age, gender, race, and PIR; Model II adjusts for age, gender, race, PIR, birthweight, and NHANES cycle; Binary logistic regression was used in the analysis. ADHD, attention-deficit/hyperactivity disorder; LD, learning disability; PIR, poverty income ratio; NHANES, National Health and Nutrition Examination Survey; OR, Odds Ratio; CI, confidence interval.

### Associations between the timing of maternal initiation of smoking cessation during pregnancy and the risk of ADHD and LD in offspring

**Figure 2:**
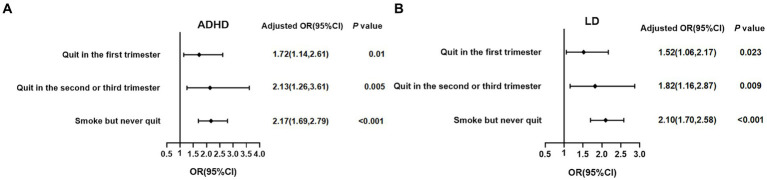
Associations between the timing of maternal initiation of smoking cessation during pregnancy and the risk of ADHD and LD in offspring. **(A)** ADHD; **(B)** LD; binary logistic regression by adjusting for age, gender, race, PIR, birthweight, and NHANES cycle was used in the analysis. The reference was non-smokers. ADHD, attention-deficit/hyperactivity disorder; LD, learning disability; PIR, poverty income ratio; NHANES, National Health and Nutrition Examination Survey; OR, Odds Ratio; CI, confidence interval.

Subsequently, we further classified the participants according to when the mothers started to quit smoking during pregnancy into four groups: non-smoker, quit in the first trimester, quit in the second or third trimester, smoke but never quit, to analyze associations with the timing of maternal initiation of smoking cessation during pregnancy with the risk of ADHD and LD in offspring. As shown in Figure 2, after adjusting for age, gender, race, PIR, birth weight, and NHANES cycle, compared to non-smokers’ offspring, maternal quitting smoking in the first trimester still posed an increased risk of ADHD (OR = 1.72, 95% CI: 1.41–2.61) and LD (OR = 1.52, 95% CI: 1.06–2.17) in offspring, and maternal quitting smoking in the second or third trimester also had a significantly increased risk of ADHD (OR = 2.13, 95% CI: 1.26–3.61) and LD (OR = 1.82, 95% CI: 1.16–2.87) in offspring. Furthermore, maternal smoking but never quitting during pregnancy had the highest risk of ADHD (OR = 2.17, 95% CI: 1.69–2.79) and LD (OR = 2.10, 95% CI: 1.70–2.58) in offspring. Interestingly, among the three groups: quitting in the first trimester, quitting in the second or third trimester, and smoking but never quitting, the risk-adjusted OR for offspring with ADHD and LD showed a gradual increase, suggesting the importance of early intervention for maternal smoking on offspring neurodevelopment.

### Sensitivity analysis

In the sensitivity analysis, excluding participants without PIR data, maternal smoking during pregnancy increased the risk of ADHD and LD in offspring compared to non-smokers’ offspring, while maternal smoking during pregnancy but quitting later still increased the risks of ADHD and LD in offspring (Supplementary Tables S2, S3). Besides, compared to non-smokers’ offspring, both maternal quitting smoking in the first trimester and maternal smoking but never quitting during pregnancy posed an increased risk of ADHD and LD in offspring. Maternal quitting smoking in the second or third trimester also had a significantly increased risk of LD (Supplementary Figure S1).

## Discussion

This study aimed to examine associations of maternal smoking cessation during pregnancy with the risk of ADHD and LD in offspring. The results showed that compared to non-smokers’ offspring, maternal smoking during pregnancy increased the risk of ADHD and LD in offspring, even if mothers later quit smoking. Furthermore, compared to non-smokers’ offspring, maternal quitting smoking in the first trimester of pregnancy still posed an increased risk of ADHD and LD in offspring.

Previous studies have found that smoking during pregnancy is associated with adverse outcomes in several areas, such as low birth weight (20), reduced intelligence (50), learning and memory deficits (51), poor academic performance (52), elevated levels of externalizing and internalizing symptoms (53, 54), and hyperactivity/attention difficulties (22). However, studies have also shown that maternal smoking during pregnancy is not significantly associated with neuropsychological functioning, intelligence (55), and the risk of ADHD in offspring (38, 56). Our study showed that compared to non-smokers’ offspring, maternal smoking during pregnancy increased the risk of ADHD and LD in offspring, even after adjusting for confounders. This finding aligns with several previous studies that demonstrated an increased risk of ADHD (27, 57, 58) and LD (29, 30) in offspring of mothers who smoked during pregnancy, supporting the idea that maternal smoking during pregnancy affects children’s neuropsychological functioning. Another study of 1,019 Finnish children born at term also observed that children of mothers who smoked before and/or during pregnancy performed worse on cognitive tests at 56 months of age than children of non-smokers (49). In summary, the varying findings of studies on maternal smoking during pregnancy and children’s neuropsychological development may be due to factors such as varying ethnicities, sample sizes, and assessment instruments. Our study suggests that maternal smoking during pregnancy may be a potential risk factor for ADHD and LD in offspring.

This study also found that the offspring of mothers who quit smoking for the rest of the pregnancy still had an increased risk of ADHD and LD compared to non-smokers’ offspring. This suggests that there is no safe period of maternal smoking during pregnancy. Similarly, Yang et al. (59) found that maternal smoking during each trimester of pregnancy significantly increased the risk of congenital anomalies at birth in offspring. One possible explanation is that the critical period of embryo development is early in pregnancy, a period that is particularly sensitive to external factors. If the mother quits smoking later, the embryo has already been exposed to harmful substances, and further exploration of the time window for quitting smoking is, therefore, crucial for the health of both the mother and the fetus. Another possible explanation is nicotine’s withdrawal effects. Concentrations of nicotine on the fetal side of the placenta generally reach levels 15% higher than maternal levels; therefore, even low levels of cigarette smoking may expose the fetus to harmful amounts of nicotine (60). Studies have shown that nicotine enhances the release of endogenous opioids (61), that newborns exposed to nicotine during fetal life become passively addicted *in utero*, and that when a pregnant woman who has regularly smoked at some point in her pregnancy quits smoking, the fetus may experience neonatal withdrawal syndrome due to a decrease in opioid levels in its tissues (62). Azuine et al. (63) showed that children exposed to opioids *in utero* are diagnosed with behavioral disorders, emotional disorders, or ADHD at approximately twice the rate of children without exposure. It was postulated that observing withdrawal symptoms in infants due to maternal smoking may predict long-term behavioral defects, such as lower intelligence and the development of ADHD (64). Besides, nicotine withdrawal effects enhance prenatal stress conditions (65), which can also have long-lasting adverse effects on the behavior and mental health of offspring (66).

We further analyzed the relationship between the timing of the mother’s initiation of smoking cessation during pregnancy and the risk of ADHD and LD in offspring. The results showed that even when mothers quit smoking in the first trimester, their offspring still had an increased risk of ADHD and LD compared to non-smokers. Similarly, a study by Räisänen et al. (21) found that infants of mothers who quit smoking in early pregnancy were slightly more likely to be admitted to neonatal care compared to infants of non-smokers. Smoking cessation in early pregnancy may not fully eliminate the risk of ADHD and LD in offspring, possibly due to smoking-related nicotine withdrawal effects mentioned above and epigenetic changes. Studies have shown that prenatal smoking can cause epigenetic changes in fetal and placental tissues, such as altered DNA methylation and dysregulated miRNA expression (67–69), which may impact the long-term health and behavior of offspring (70, 71). Notably, smoking-related epigenetic effects could potentially be transmitted across generations (i.e., smoking influences may affect germline cells or escape epigenetic reprogramming during development) (70, 72). Therefore, even if smoking cessation happened early in pregnancy or before conception, offspring would still be at risk of poor outcomes. Additionally, there is a half-life for hazardous substances contained in tobacco, as shown in Table 4 (73), with one publication reporting a maximum half-life of 6 weeks for biomarkers of tobacco exposure. If a pregnant woman smokes daily before pregnancy, tobacco accumulates in her body; therefore, even if she quits smoking in early pregnancy, the accumulated toxins in the body have not yet been fully metabolized, and these can pass through the bloodstream into the placenta, affecting the embryo’s brain development. As in the study by Heinonen et al. (49), they found that children whose mothers smoked >10 cigarettes daily and quit before pregnancy had significantly lower cognitive scores than children of never-smokers. Finally, our study showed a gradual increase in the risk-adjusted ORs for ADHD and LD in three groups: quitting in the first trimester, quitting in the second or third trimester, and smoking but never quitting. This indirectly reflects a possible dose-dependent association between maternal smoking and the risk of ADHD and LD in offspring. Our study also supports the speculation of Zou et al. (74) that exposure to tobacco throughout pregnancy is more associated with brain development than transient exposure in early gestation. Further studies are needed to replicate our findings and reveal the mechanisms behind these clinical findings. We believe that these findings have important public health implications, indicating the need to focus on pre-pregnancy healthcare and smoking cessation support for women to promote healthy brain development in children.

**Table 4:** Biomarkers of tobacco exposure.

Biomarker	Precursor	*t*½
Nicotine	Nicotine	1–2 h
Cotinine	Nicotine	16–18 h
Anatabine	Anatabine	10–16 h
NNAL, NNAL-glucuronides	NNK (TSNA)	6 week
Exhaled CO	Carbon monoxide	2–6 h
Carboxyhemoglobin	carbon monoxide	4–6 h
1-hydroxypyrene and other polycyclic aromatic hydrocarbon (PAH) metabolites	PAHs	20 h
Mercapturic acid metabolites	1,3-Butadiene	–
Mercapturic acid metabolites	Acrolein	–
Acetonitrile	Acetonitrile	32 h
*S*-phenyl-mercapturic acid	Benzene	9 h
Thiocyanate	Hydrogen cyanide	7–14 days

NNAL, 4-(methylnitrosamino)-1-(3-pyridyl)-1-butanol. NNAL-gluc, 4-(methylnitrosamino)-1-(3-pyridyl)-1-butanol glucuronide; NNK, 4-(methylnitrosamino)-1-(3-pyridyl)-1-butanone; TSNA, tobacco-specific nitrosamines; PAH, polycyclic aromatic hydrocarbons.

However, this study had some limitations. First, other risk factors (e.g., gene–environment correlations or maternal health problems) may affect the risk of ADHD and LD; however, we could not adjust for these because such data are not included in the NHANES database. Therefore, we could not establish a causal relationship between maternal smoking and the risk of ADHD and LD in offspring. Second, there was heterogeneity in tobacco exposure among mothers who smoked during pregnancy. However, the NHANES database does not include the frequency of maternal smoking during pregnancy, and if the mother was usually a heavy smoker, then even if she had quit smoking during pregnancy, the significance of quitting smoking during pregnancy on the health of the offspring may not be fully revealed. Finally, our study’s smoking and smoking cessation variables were based on self-reported data, leading to potential subjective biases that should be considered when interpreting the results. Future studies need to integrate genetic background and maternal health issues, use objective indicators, and conduct prospective cohorts to further explore the effects of smoking, smoking cessation, and the timing of smoking cessation.

## Conclusion

We found that maternal smoking during pregnancy increased the risk of ADHD and LD in offspring, even with subsequent cessation. Furthermore, maternal smoking cessation early in pregnancy may still increase the risk of ADHD and LD in offspring compared to non-smokers’ offspring. It seems that the later the mothers quit smoking during pregnancy, the higher the risk of ADHD and LD in offspring. This study has public health implications: women should quit smoking as early as possible in pregnancy or even in preparation for pregnancy, and physicians should provide health guidance, including smoking cessation support and treatment, to women planning a pregnancy to maximize healthy growth and optimal brain development in their offspring.

## Data availability statement

Publicly available datasets were analyzed in this study. This data can be found here: https://www.cdc.gov/nchs/nhanes/index.htm.

## Ethics statement

The studies involving humans were reviewed and approved by The NCHS Research Ethics Review Board. The studies were conducted in accordance with the local legislation and institutional requirements. Written informed consent for participation in this study was provided by the participants’ legal guardians/participants.

## Author contributions

QL: Conceptualization, Formal analysis, Writing – original draft, Writing – review & editing. XC: Funding acquisition, Writing – review & editing. HZ: Writing – review & editing. DM: Funding acquisition, Writing – review & editing. NL: Conceptualization, Writing – review & editing.

## Footnotes

^1^ https://wwwn.cdc.gov/nchs/nhanes/Default.aspx
