# Supplementary figures and images for: Maternal smoking cessation in the first trimester still poses an increased risk of attention-deficit/hyperactivity disorder and learning disability in offspring
Source: Front Public Health. 2024 Jul 16;12:1386137. doi: 10.3389/fpubh.2024.1386137 (PMC11286595; doi:10.3389/fpubh.2024.1386137)

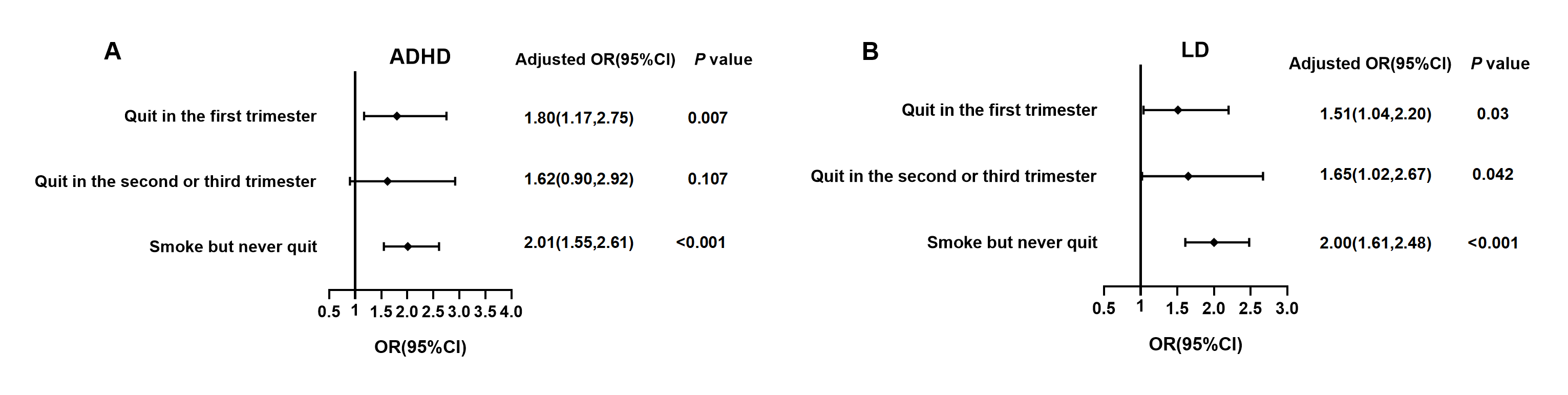

Supplement: Supplementary file 4 [file Image_1.TIF]
